# Clonal evolution of *Candida albicans, Candida glabrata* and *Candida dubliniensis* at oral niche level in health and disease

**DOI:** 10.1080/20002297.2021.1894047

**Published:** 2021-03-15

**Authors:** Alexander J. Moorhouse, Rosa Moreno-Lopez, Neil A.R. Gow, Karolin Hijazi

**Affiliations:** a Institute of Medical Sciences, School of Medicine Medical Sciences and Nutrition, University of Aberdeen, Foresterhill, Aberdeen, UK; b School of Cellular and Molecular Medicine, University of Bristol, Bristol, UK; c Department of Genetics and Genome Biology, University of Leicester, Leicester, UK; d Institute of Dentistry, School of Medicine Medical Sciences and Nutrition, University of Aberdeen, Foresterhill, Aberdeen, UK; e Medical Research Council Centre for Medical Mycology at The University of Exeter, University of Exeter, UK

**Keywords:** Oral niche, *Candida albicans*, *C. glabrata*, *C. dubliniensis*, MLST, strain type, clonal evolution

## Abstract

**Background:** *Candida* species have long been recognised as aetiological agents of opportunistic infections of the oral mucosa, and more recently, as players of polymicrobial interactions driving caries, periodontitis and oral carcinogenesis.

**Methods:** We studied the clonal structure of *Candida* spp. at oral niche resolution in patients (n = 20) with a range of oral health profiles over 22 months. Colonies from oral micro-environments were examined with multilocus sequencing typing.

**Results:** *Candida* spp. identified were *C. albicans, C. glabrata* and *C. dubliniensis*. Increased propensity for micro-variations giving rise to multiple diploid strain types (DST), as a result of loss of heterozygosity, was observed among *C. albicans* clade 1 isolates compared to other clades. Micro-variations among isolates were also observed in *C. dubliniensis* contra to expectations of stable population structures for this species. Multiple sequence types were retrieved from patients without clinical evidence of oral candidosis, while single sequence types were isolated from oral candidosis patients.

**Conclusion:** This is the first study to describe the clonal population structure, persistence and stability of *Candida* spp. at oral niche level. Future research investigating links between *Candida* spp. clonality and oral disease should recognise the propensity to micro-variations amongst oral niches in *C. albicans* and *C. dubliniensis* identified here.

## Introduction

The oral cavity comprises a diverse set of niches inhabited by diverse microbial colonisers of prokaryotic (bacteria) and eukaryotic (yeasts and filamentous fungi) microorganisms. Distinct micro-environments harbour microbial communities of planktonic cells in salivary fluids, and of adherent and filamentous multi-species biofilms growing on the surface of teeth, the tongue and mucosal membranes [1,2]. Multi-species and cross-kingdom co-aggregations among complex biofilms contribute to ecological homeostasis during host health, and are important factors in the transition to dysbiosis and disease progression [3]. As such most oral diseases are caused by dysbiotic shifts within the microbial communities where ‘pathobionts’ such as *Candida* spp. thrive quantitatively and functionally.

*Candida* spp. are the main aetiological agent of oropharyngeal mycoses that present in patients with predisposing systemic factors such as immune deficiencies as well as local factors such as denture wearing, smoking and salivary hypofunction. In addition, *Candida* spp. contribute to the cariogenic process acting synergistically with *Streptococcus mutans* to increase adherence properties which promotes community expansion and causes tissue damage [4,5]. *Candida* spp. are also prevalent in the periodontal environment – a synergistic role similar to that occurring within cariogenic biofilms is hypothesised although no mediating mechanism has yet been confirmed [6,7].

Molecular epidemiology and study of the population structure of *Candida* spp. in the oral cavity develops our understanding of their role in oral health and disease. Multi-Locus Sequence Typing (MLST) is largely accepted as the standardised measure of population structure for *Candida* spp. [8–13]. Notwithstanding that 18S ribosomal DNA subunit and whole-genome sequencing are also usefully applied [14], MLST has been instrumental in defining the accepted model of *C. albicans* population structure as one of predominant clonality with rare recombination events as a result of cryptic mating strategies generating new highly differential strain types, and more often, incremental mutation events such as point mutations and loss of heterozygosity (LOH) providing low level, within population, genetic diversity [15–21]. Stability and plasticity among *C. albicans* populations using serial isolate MLST studies conducted over several months and years revealed micro-variations associated with the emergence of resistant phenotypes [22,23]. A more marked clonality among molecular types with highly stable population structure has been observed among commonly isolated, yet less pathogenic, non-albicans *Candida* spp. such as *C. dubliniensis* [9], *C. glabrata* [24], and *C. tropicalis* [25], although micro-variation amongst sequential clonal isolates has been reported in these *Candida* spp [26–28].

Enrichment and stability of species and strain types among colonising populations of *Candida* spp. causative of specific infection types have been highlighted by MLST studies [6,24,29]. *C. albicans* is the major cause of oral fungal infections and readily forms mixed species biofilms with partner *Candida* spp. of more clonal population structures, such as with *C. glabrata* in denture stomatitis lesions [30], or with *C. dubliniensis* in HIV patients [31]. Enrichment of *C. albicans* DSTs has been reported among epidemiological groups, for example, of clade 1 DSTs among isolates from periodontitis patients [6]. However, such correlations are often not always reproducible [32]. *C. albicans* DSTs are frequently varied within population groups; for example, among isolates from childhood caries cases [33]. Moreover, *C. albicans* micro-variations, which may arise during the course of serial sampling studies [23,29], give rise to new DSTs which contribute an inherent dynamic temporal instability to the phylogenetic topologies that define strain type clades for *Candida* species.

We set out to assess the persistence and stability of *Candida* spp. and MLST strain types amongst oral micro-environments of a cohort of 20 dental patients comprising patients suffering from oral mucosal candidosis and participants with no history of oral candidosis. Sampling strategy was designed to allow detection of population strain type variation and evolution of antifungal resistance within the same patient by sequencing several colonies from multiple oral sites for each patient and repeating sampling multiple times where possible within a 2-year period.

To the best of our knowledge, this is the first longitudinal study of multiple *Candida* spp. through MLST at the oral niche level.

## Materials and methods

### Patient recruitment and sampling

**Ethical consent**: Ethical approval for involvement of human participants in this study was granted by North of Scotland Research Ethics Service (REC reference number 12/NS/0006). Written informed consent was obtained from all subjects according to the World Health Organisation guidelines for Good Research Practice.

**Patient recruitment**. Participants were recruited from patients attending routine clinics at the University of Aberdeen Dental Hospital. Oral candidosis and periodontitis cases were diagnosed using accepted clinical criteria [34,35]. Patient assessment included a full history, neck and oral mucosae examination, tooth charting, periodontal health assessment, measurement of plaque index and whole salivary flow rate. The sample cohort consisted of 20 dental patients with a range of oral and general health statuses including 8 participants without oral mucosal candidosis and 12 patients with oral candidosis and a range of predisposing factors. Clinical characteristics of the patient cohort are reported in Supplemental Table S1.

**Figure 1.**
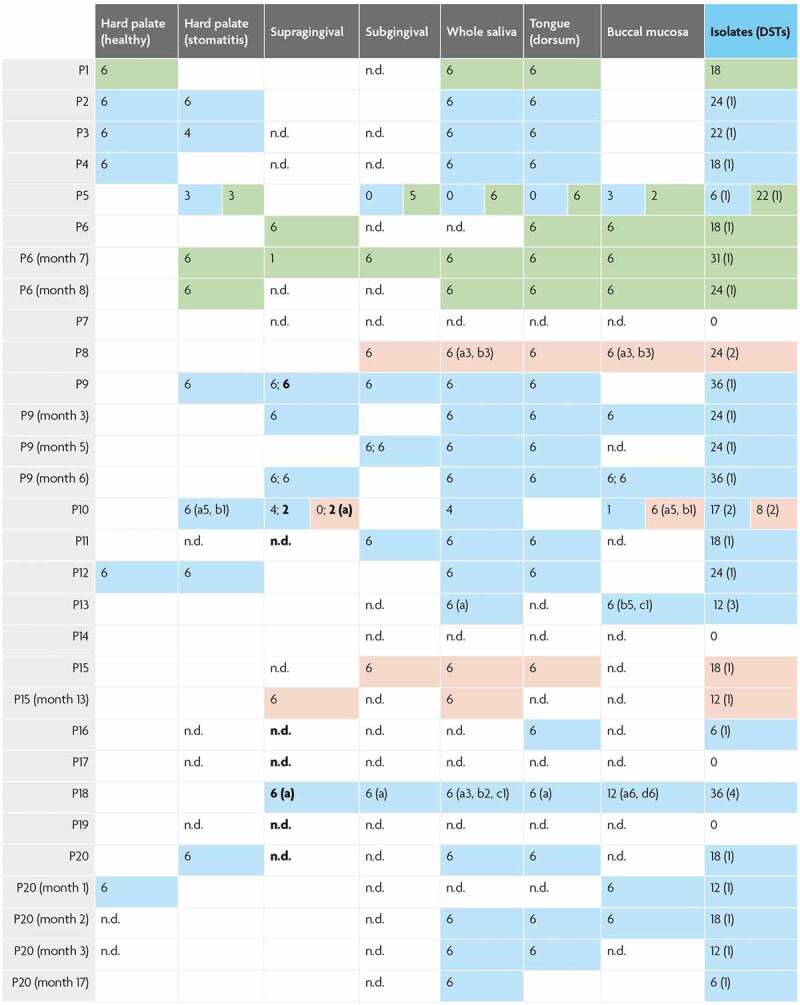
*Candida* spp. colonies typed from oral niches

**Sampling strategy**: Patients samples were collected from the following oral micro-environments: plaque and calculus from subgingival and supragingival sites, unstimulated whole saliva samples, tongue dorsum scrapings, swabs from buccal and palatal mucosa (both healthy and inflamed sites in oral candidosis patients). Swabs were stored in Amies medium for transportation of aerobes and anaerobes (MWE, Wiltshire, England). Plaque, calculus, scrapings and swabs were suspended in phosphate-buffered saline (PBS). Samples were transported and stored at 4°C prior to downstream experiments within the same working day. Serial sampling from three patients was conducted over 1–17 month periods. As shown in Figure 1 the sampling effort from different oral sites amongst the cohort of 20 patients was slightly uneven: palate 65%, supragingival 65%, subgingival 85%, saliva 100%, dorsum of tongue dorsum 95%, and buccal mucosa 75%. Frequency of the niche sampling relative to total sampling events was as follows: palate 60%, supragingival 60%, subgingival 83%, saliva 100%, dorsum of tongue 93%, and buccal mucosa 77%.

### *Species identification of* Candida *spp. isolates*

Patient samples were plated and purified on Sabouraud dextrose (Sabdex) agar plates containing 1% mycological peptone (w/v), 4% (w/v) glucose and 2% (w/v) agar with chloramphenicol (50 μg/mL) and gentamycin (20 μg/mL) and incubated at 30°C, for 48 h and checked at 24 h for growth. Enrichment of lower abundance sample types (plaque and calculus) were inoculated to YPD broth cultures (1% yeast extract, 2% bacto-peptone and 1% glucose) with chloramphenicol (50 μg/mL) and gentamycin (20 μg/mL) and incubated at 30°C, for 48 hrs and checked at 24 h for growth. Aliquots of YPD enrichment cultures were plated and purified on Sabdex agar, incubated at 30°C for 48 h and checked at 24 h for growth. Six colonies were selected from separate areas of culture positive Sabdex plates and grown in YPD at 30°C overnight for preparation of glycerol stocks (stored at −80°C) and genomic DNA. CHROMagar Candida chromogenic growth media [36] and ITS diagnostic PCR product sequencing [37] were used as previously described for species identification prior to sequencing isolates for MLST.

### Antifungal susceptibility testing

Minimum inhibitory concentrations were determined by broth micro-dilution testing following the EUCAST and CLSI guidelines [38,39]. The drug concentrations ranged from 0.032 mg/ml to 16 mg/ml for caspofungin and amphotericin B, and 0.13 mg/ml to 64 mg/ml for fluconazole and voriconazole. Each drug was serially diluted with sterile water to 50 μl in flat bottomed 96 well plates. Cultures were grown in NGY medium (0.1% (w/v) neopeptone, 0.4% (w/v) glucose, and 0.1% (w/v) yeast extract) for 12 h at 30°C until exponential phase, and 20 μl were transferred to 11 ml RPMI inoculation medium (2 x RPMI-1640, 2% (w/v) glucose, buffered to pH 7.0 with MOPS). Aliquots of 50 μL inocula in RPMI were added to drug plates (total volume 100 μL), incubated for 24 h at 37°C and analysed using a VERSAmax microplate reader at 405 nm. Three colonies of each sequence type from each patient, at each sample time point were assayed in triplicate technical replicates and the experiment repeated for confirmation.

### Genomic DNA extraction

Pelleted overnight liquid YPD cultures were transferred to 2 mL Eppendorf tubes and vortexted for 5 min in the presence of 0.3 g of acid washed glass beads, 200 μL extraction buffer (2% Triton X-100, 1% SDS, 100 mM NaCl, 10 mM Tris pH 8.0, 1 mM EDTA), 200 μL phenol: chloroform alcohol (1:1). TE buffer (200 μL, Tris EDTA pH 7.5) was added and the tubes inverted. Phase separation by centrifugation for 5 min at 14,000 g was followed by transfer of the aqueous layer to 1 mL of 100% EtOH in 2 mL tubes and centrifugation for 2 min at 14,000 g to obtain precipitated DNA. Pelleted DNA was resuspended in 400 μL TE plus 10 μL RNase A (10 mg ml-1) and incubated for 20 min at 37°C, subsequent precipitation in 20 μL 3 M NaOAc and 1 mL 100% EtOH with centrifugation at 14,000 g for 10 min was followed by resuspension in 200 μL sterile H_2_O, as previously described [40].

### PCR and sequencing

PCR reactions were performed in 20 uL volumes in 96 well microtitre plates using high-fidelity DNA polymerase (Finnzymes Phusion F-530 L) according to the manufacturer’s instructions with first cycle denaturation for 2 min at 94°C, followed by 25 cycles of denaturation at 94°C for 1 min, annealing at 52°C for 1 min, elongation at 72°C for 1 min, and a final extension step of 10 min at 72°C and as previously described [12]. The PCR product (5 μL) was purified using Mag-Bind E-Z Pure solution and eluted in 20 μL of sterile H_2_O according to the manufacturer’s instruction.

Bi-directional sequencing reactions were performed as previously described [14,41,42] in 5 μL volumes comprising 1 μL of purified PCR product, 2 μL diluted primer (1:15 dilution, with sterile H_2_O), 2 μL BigDye cocktail (for 200 samples 50 μL Big Dye, 175 μL 5 x Buffer, 175 μL sterile H_2_O) in 96 well microtitre plates. The reactions proceeded for 25 cycles of 96°C for 10 sec, 52°C for 5 sec, 60°C for 2 min and held at 10°C. The products were purified by addition of 10 μL sterile H_2_O to prevent dye blobs, and 50 μL precipitation solution (48 μL 100% EtOH, 2 μL 3 M NaOAc pH 4.6). Incubation for 45 min at RT was followed by centrifugation at 4°C for 1 h at 2,254 g, washing in 150 μL 70% EtOH with 10 min centrifugation 2,254 g at 4°C, drying for 2 min at 80°C and storage at −20°C until dispatch for sequencing. Sequencing was performed on ABI 3730 instruments by the Zoology Department of the University of Oxford (Oxford, UK).

### Phylogenetic analysis

DNA sequence results were analysed and SNPs determined using Lasergene SeqMan Pro software by DNASTAR (https://www.dnastar.com/). Strain types were further confirmed by subsequent re-sequencing using genomic DNA freshly prepared from cultures grown from stored −80°C glycerol strain stocks. DSTs were submitted to the *C. albicans* (http://calbicans.mlst.net/) and *C. glabrata* (http://cglabrata.mlst.net/) MLST central curation databases. Multiple sequence alignment and tree building with the Unweighted Pair Group Method with the Arithmetic Mean (UPGMA) method was performed using Clustal Omega [43]. Splits phylogenies were prepared from diploid sequence type (DST) profiles of *C. glabrata* and *C. albicans* in SplitsTree4 v4.13.1 [44]. eBurst analysis was performed with eBURSTv3 [45]. Beast was used with Beauti, Tree Annotator and LogRecombiner to create ML trees including *C. dubliniensis* sequences and visualised using FigTree v1.4.0 [46–48]. PHASE [49], was used to infer phased haplotypes for micro-variants, and variable base position frequencies for micro-variant populations were visualised with WebLogo 3 [42].

## Results

### Candida *spp. and clonal distribution within the study cohort*

The study cohort consisted of 20 dental patients with a range of oral and general health statuses including 8 control subjects without clinically diagnosed candidosis (P4, P6, P7, P8, P13, P14, P15 and P18) and 12 patients with oral mucosal candidosis Table S1). MLST sequence analysis was conducted for 369 *C. albicans* isolates, 113 *C. glabrata* isolates and 68 *C. dubliniensis* isolates cultured from clinical samples collected from 6 oral niches. Six out of eight patients without clinical oral candidosis were positive for *Candida*. spp., consistently with carriage levels cited among healthy individuals [50]. Of these six patients, three patients yielded *C. albicans*, 1 *C. glabrata* (P6, a partial denture-wearer) and 2 *C. dubliniensis* (P15 and P8 non-denture wearers with periodontitis) (Figure 1, Table S1). Of four non-denture-wearing participants without oral mucosal candidosis, only one proved negative for *Candida* spp. (P14) (Figure 1, Table S1).

Ten out of 12 patients diagnosed with oral mucosal candidosis yielded *Candida* spp. (P10, P11, P16, P1, P2, P3, P5, P9, P12, P20). All oral candidosis patients yielded *C. albicans* except for P1 who yielded *C. glabrata* alone. An HIV-positive patient with severe periodontitis and partial dentures yielded *C. albicans* in co-occurrence with *C. dubliniensis* (P10) while another patient yielded *C. albicans* with *C. glabrata* (P5) (Figure 1, Table S1).

The majority of sequence types in this study were retrieved from two patients presenting without oral candidosis clinically: P18 (partial denture-wearer with severe periodontitis) and P13 (non-denture wearer with type 1 diabetes). Patients yielding a single DST from clade 1 *C. albicans* presented with clinical oral candidosis without periodontitis. Both patients yielding *C. albicans* with *C. glabrata* (P5) and with *C. dubliniensis* (P10) yielded clade 1 *C. albicans* isolates. *C. albicans* isolates not in clade 1 were retrieved exclusively from patients presenting with clinical oral candidosis with the exception of one patient (P4; smoker, denture-wearer, severe periodontitis) (Figure 1, Table S1).

Diagram showing number of colonies at the oral niche level from the study cohort. Patient unique identifiers as well as serial sampling events per patient are enumerated vertically. The number of colonies obtained for each of the 6 oral niches is indicated in each row with colour-coding for each *Candida* species as follows: green cells = *C. glabrata* isolates; light blue cells = *C. albicans* isolates; pink cells = *C. dubliniensis* isolates. Blank cells = sites not sampled; n.d. = no isolates detected. Instances where sub-/supra-gingival sites were sampled more than once are indicated in the same cell and separated by a semi-colon. Isolates retrieved from calculus are indicated in bold. Where more than one diploid sequence type (DST) was identified, ‘a, b, c’ is marked in parenthesis along with the number of colonies for each DST that were obtained. The total number of DSTs identified is given in parenthesis.

### Candida *spp. at oral niches*

Of 20 patients sampled, 80% were positive for *Candida* spp. In respect of niches sampled from *Candida* spp. positive participants, saliva and tongue surface samples yielded isolates most frequently (83% and 81% positive), while supra-gingival and sub-gingival samples yielded least often (56% and 38% positive), and buccal mucosal and hard palate swabs were positive for *Candida* spp. in 67% and 68%, respectively. Higher retrieval of *Candida* isolates from tongue and saliva should be viewed in the context of a slightly higher sampling frequency from tongue and saliva as compared to other sites (Figure 1). Sub-gingival samples yielded *Candida* isolates least frequently despite a higher sampling frequency for this site as compared to palate, buccal mucosa and supra-gingival plaque.

**Figure 2.**
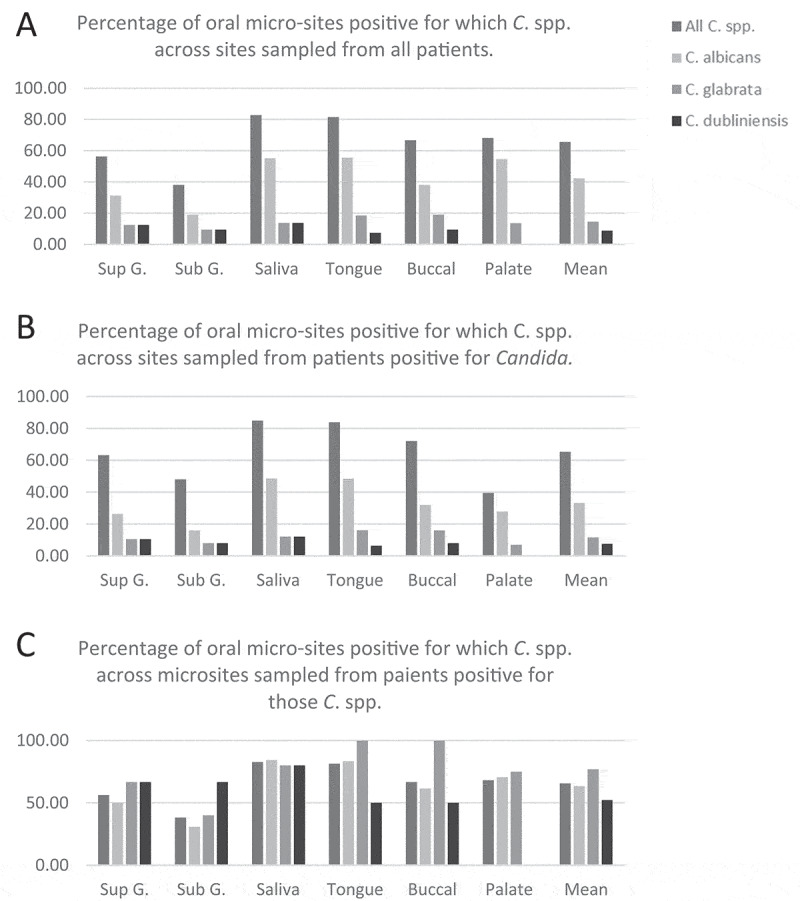
Summary of *Candida* spp. presence at oral niches

*C. albicans* was the most abundantly isolated (369 isolates) accounting for 67% of typed colonies. *C. albicans* was also the most frequently present *Candida* spp. across the niches sampled, in respect of niches sampled among all participants (33%) as well as across *Candida* spp. positive participants (42%); with *C. glabrata* (12% and 15%) and *C. dubliniensis* (8% and 9%) second and third, respectively (Figure 2 A-B). Non-*albicans Candida* spp. were identified in six participants; three yielded *C. glabrata* (including one as co-present with *C. albicans*) and three participants yielded *C. dubliniensis* (including one as co-present with *C. albicans*). Across all patients, each of these three species were found at least once at each of the niches sampled, with the exception of the hard palate which did not yield *C. dubliniensis*.

The proportion of *Candida* spp. positive niches from patients positive for each specific *Candida* spp., was highest for *C. glabrata* (77%), owing largely to 100% isolation from the tongue surface and buccal mucosa sites (Figure 2C). *C. dubliniensis* isolation was the most consistently retrieved species at gingival sites (67%), but less consistent over all niches (52%) than *C. albicans* (63%) (Figure 2C). Both *C. glabrata* and *C. dubliniensis* were more consistently retrieved across all niches among patients also yielding *C. albicans*.

The number of colonies and frequency of isolation of *Candida* species sampled from 6 oral niches of 20 patients is given. Sites above and below the gingival margin are Sup.G and Sub.G, respectively, other sites are whole saliva, the dorsum of the tongue, the hard palate and the buccal mucosa.

### *Population structure of* Candida *spp.*

***C. albicans* population structure at oral niche level**. Of the 17 *C. albicans* DSTs we identified, only 7 had been reported previously (Table 1). A new sequence type for the ZWF1a region was identified from a clade 2 P4 isolate and designated ZWF1a ST 255 through the central *C. albicans* MLST curation service. ZWF1a ST 255 was distinguished from the closest matching sequence ST 4 by the 47th base of the sequence being heterozygous R rather than homozygous G.

**Table 1.** *C. albicans* MLST DSTs for typed colonies. Diploid sequence types (DSTs) for *C. albicans* isolates showing the sequence type numbers for each sequenced region and clade membership and the number of colonies sequenced. DSTs have been submitted to the central MLST database; newly identified DSTs are highlighted green

Patient	*N*	AAT1a	ACC1a	ADP1	MPIb	SYA	VPS13	ZWF1b	DST	Clade
P2	24	5	32	21	34	7	55	5	**732**	3
P3	22	2	3	5	2	2	20	5	**237**	1
P4	18	13	2	4	4	4	4	255	**2422**	2
P5	6	2	77	5	31	2	3	5	**2118**	1
P9	120	13	10	15	6	7	55	12	**2119**	3
P10(a)	16	2	3	5	2	2	21	5	**110**	1
P10(b)	1	2	5	5	2	2	21	5	**37**	1
P11	18	4	17	21	19	27	32	22	**88**	12
P12	24	4	7	4	4	4	26	4	**497**	2
P13(a)	1	11	5	5	4	2	20	5	**2423**	1
P13(b)	5	31	5	5	2	2	21	5	**375**	1
P13(c)	6	120	5	5	4	2	20	5	**2126**	1
P16	6	4	2	4	4	34	4	204	**2120**	2
P18(a)	27	31	5	5	2	2	21	5	**375**	1
P18(b)	2	31	3	5	2	2	21	5	**2122**	1
P18(c)	1	31	5	5	2	2	24	12	**2131**	1
P18(d)	6	31	5	5	2	2	21	12	**2424**	1
P20	66	71	13	6	1	7	218	15	**2123**	8

Isolates from clade 1 were the most frequent among the isolates. This was also the only clade to contain multiple isolates from individual participants. DSTs from 5 of the 20 patients comprised 10 clade 1 DSTs, 4 of which had been previously reported; DST 88 and DST 497 (P10), DST 273 (P3), and the only DST identified in more than one patient (P13 and P18) – DST 375. Multiple *C. albicans* DSTs were identified from sample material from each of three patients: P10 (2 DSTs), P13 (3 DSTs) and P18 (4 DSTs) – all clade 1 isolates. From P3 and P5 two further clade 1 DSTs were retrieved (DST 237 and DST 2118, respectively) (Table 1).

**Figure 3.**
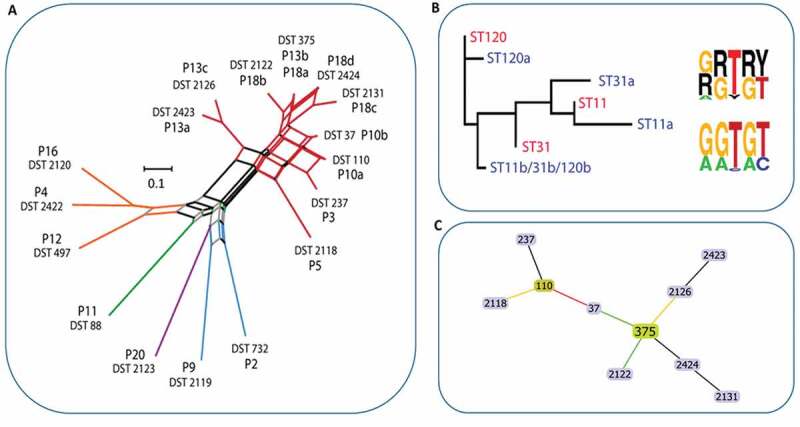
Population structure of *C. albicans* isolates. **A**) Phylogenetic network of *C. albicans* DSTs. Branches are coloured by clade as follows: 2, yellow; 12, green; 8, purple; 3, blue; 1, red; branches are grey where splits are shared. **B**) Cladogram (left) of P18 AAT1a microvariant sequences including the unphased ST (red) and both phased deconvolved sequences (blue) and nucleotide motif logos (right) for the unphased ST nucleotide variants (top) and phased haplotypes (bottom). **C**) eBURST analysis of *C. albicans* DSTs identified. The eBURST colouring convention for nodes is: founder, lime green; sub-founder, olive green; and for connecting line colours it is: high-level edges, gold; triple locus variant, red; double locus variant, green; branches are black and were without recourse to tiebreak rules

The average pairwise distance of clade 1 isolates (0.39 between all, 0.4 between isolates from separate patients) was lower than that of other clades for which multiple DSTs were identified, clade 2 (0.52) and clade 3 (0.71). The clade 2 (3 DSTs) and clade 3 isolates (2 DSTs) each included a single previously identified DST (DST 497 and DST 732, respectively), the remaining previously identified DST (DST 88 from P11) belonged within clade 12. The final remaining DST 2123, a clade 8 isolate, was identified in a patient presenting with widespread and severe erythematous candidosis secondary to long-term use of broad-spectrum antibiotics (P20) and had not been previously reported. P13 and P18 isolates comprised 35% of all DSTs and 60% of clade 1 DSTs, 3 sequence types for each of AAT1a and VPS13, and 2 for ACC1a. The MP1b and ZWf1a regions were identified for these DSTs. Determination of the haplotype phase for these micro-variant isolates revealed that micro-variant DSTs consisted of variant types for ACC1a, MP1b and ZWf1a that were distinguishable from DST 375 in being homozygous at a series of consecutive SNPs – a pattern of LOH (Figure 3 and Figure S1).

Phylogenetic analysis of AAT1a and VPS13 independently including also their computationally phased variant counterpart sequences, demonstrated that DST 375 commanded the least average distance from each of the phased micro-variant sequences. Together with eBURST analysis of DST profiles these data indicated that clade 1 isolates identified within this study comprised micro-evolutionary changes across the sequenced regions as a result of LOH events which also occasionally involved additional point mutations (AAT1a and VPS13) stemming from the progenitor DST 375 (Figure 3 and Figure S1).

**Figure 4.**
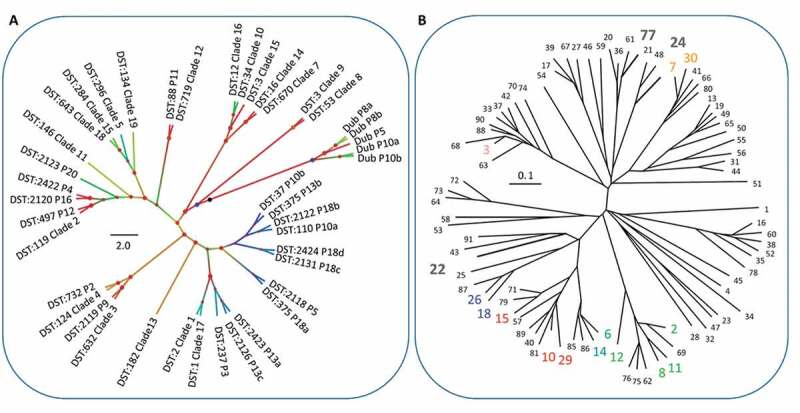
Population structure of *C. dubliniensis* and *C. glabrata* isolates. **A**) Phylogenetic tree of *C. albicans* and *C. dubliniensis* isolates identified including also representative strain types of the major *C. albicans* clades. The tree is produced from MLST sequences using the maximum likelihood method and is rooted at the black node, branches are gradient coloured bootstrap probabilities (red-high, blue-low), and nodes are sized by bootstrap support and coloured by branch length (red-short, blue-long). **B**) Neighbour joining phylogeny of the phylogenetic tree of *C. glabrata* isolates identified (grey bold type) including also all isolates present in the MLST database at the time of production (small black and coloured type). The tree is produced from MLST sequences using the neighbour joining method. STs representative of major groups are coloured: Group 1, pink; Group 2, blue; Group 3a, red; Group 3b, blue-grey; Group 5, green)

***C. glabrata* and *C. dubliniensis* population structure at oral niches**. *C. dubliniensis* sequence types were also distinct between participants, two distinct sequence types were identified from both P10 and P8, and a single sequence type identified from P15 (Figure 1). Currently, *C. dubliniensis* is without a centrally curated MLST database. Thus, no sequence type numbering system is applicable. Previous studies have implemented varied primer combinations usually including several regions included in the closely related *C. albicans* MLST scheme and the addition of RPN2 to reinforce discriminatory power [9,40,51]. The six core MLST regions shared directly with the *C. albicans* MLST scheme (ACC1a, ADP1, MP1b, SYA, VPS13 and at the ZWF1 region) identified 26 SNPs that distinguished 5 sequence types. Nine SNPs separated two P8 sequence types, and 5 SNPs separated two P10 sequence types – interestingly in both cases, only one SNP was a heterozygous/homozygous mutation. Phylogenetic analysis confirmed within-patient monophyly for micro-variant *C. dubliniensis* isolates from P8 and P10 (Figure 4).

Each of the three *C. glabrata* MLST sequence types (STs) identified in this study have been reported in previous studies, and are from distinct clonal clusters (CCs). A single ST was identified for each of three patients, P1 (ST 22, CC 15), P5 (ST 24, CC 52), P6 (ST 77, CC 77). *C. glabrata* (ST24, CC52) and *C. albicans* (DST 2118, clade 1) isolates were retrieved from P5. *C. glabrata* STs remained clonal for all three patients with no evidence of micro-variation amongst niches or sample time points (Table 2 and Figure 4).

**Table 2.** *C. glabrata* MLST STs for typed colonies. Sequence types (STs) determined by MLST for *C. glabrata* isolates showing the sequence type numbers for each sequenced region and clade membership and the number of colonies sequenced. STs have been submitted to the central MLST database

Patient	N	FKS	LEU2	NMT1	TRP1	UGP1	URA3	Sequence type	Clonal cluster
P1	18	7	5	6	12	1	8	22	15
P5	22	9	9	10	4	3	2	24	52
P6	73	10	14	12	11	3	2	77	77

### Antimicrobial susceptibility

Antifungal resistance profiles for these isolates were generally unremarkable, with no differences identified between isolates of the same species from the same patient that would otherwise indicate the development of resistance during the course of the study (data not shown). Minimum inhibitory concentrations were calculated as MIC 90 values for caspofungin, AmBisome and fluconazole on a per patient for *C. albicans, C. glabrata* and *C. dubliniensis* basis rather than for each sequence type. No changes in MIC resulted in the susceptibility exceeding the reported break-points for specific *Candida* species and specific antifungal agents (data not shown).

### Discussion

This study employed a high-resolution sampling strategy to examine multiple colonies from multiple oral niches sampled from a clinically diverse cohort at multiple time points over 22 months. Specifically, this approach has the potential to identify micro-variants directly associated with specific phenotypes. Although micro-evolutionary changes had taken place among isolates from the same patient, these did not coincide with any phenotypic advantage in terms of antifungal resistance contra to findings of a study of 43 isolates of *C. albicans* from 11 HIV patients with oro-pharyngeal candidosis [52].

Our study identified *C. albicans* as the predominant *Candida* spp. present with clade 1 being the most represented. Enrichment for *C. albicans* MLST clade 1 isolates with untreated periodontitis was previously reported [9]. Indeed, clade 1 isolates have emerged as the most commonly encountered clade among MLST sequence types generally [53,54].

There were a larger number of oral micro-niches colonised by and a larger number of strain types observed for *C. dubliniensis* isolates when present along with *C. albicans*. Equally, *C. glabrata* was more consistently retrieved across all niches among patients also yielding *C. albicans*. This suggests cooperation between these species in forming biofilm, rather than competitive colonisation, and potentially in generating a greater diversity of oral niches compared with mono-species colonisation. The increased prevalence of *C. albicans* and *C. glabrata* in denture stomatitis has been observed in several previous studies [30,55–57] and their presence as dual species biofilm was highlighted [30]. The prevalence of *C. dubliniensis* and its association with HIV [31,58] and periodontitis has also been identified in several previous studies [58–60], and its co-occurrence with *C. albicans* was also highlighted [59]. Other *Candida* spp. which are often reported at higher prevalence than *C. dubliniensis* [7,55], and among denture stomatitis patients, e.g. *C. krusei* and *C. parapsilosis* [56,61], or in oral samples in general, e.g. *C. tropicalis* [61,62], were not observed in this cohort.

We showed that micro-variations in *C. albicans*, as a result of LOH events and occasional point mutations, were responsible for the presence of multiple DSTs within individual patients and oral niches. The reduced pairwise phylogenetic distance and increased propensity for micro-variation observed among *C. albicans* clade 1 isolates was consistent with propositions of an increased ability to evolve and reduced evolutionary constraint for clade 1 isolates compared to other clades. Future studies should consider the potential role of recombination events through the para-sexual cycle in the evolutionary ability of clade 1 in addition to LOH events, in light of evidence demonstrating the importance of gene flow in the diversification of *C. albicans* clades [63]. Genetic diversification through micro-variation has been associated with exposure to adverse environmental conditions [64]. On the other hand, the possibility that varying environmental conditions in healthy and infective states may themselves favour recurrent colonization by different clones should be considered.

We observed marked clonality and stability among *C. glabrata* isolates in keeping with previous evidence [24,65,66], notwithstanding recent data suggesting the potential phenotypic importance of genetic variations within infecting clonal populations [67]. On the other hand, we observed micro-variation among isolates of *C. dubliniensis* from individual patients contra to expectations of highly clonal population structures for this species [9,31,68].

In this study cohort, we did not find evidence of co-colonisation in the same patient by more than one lineage of any of the three species. Co-infection by independent lineages of *C. parapsilosis* was recently reported in a patient with post-surgical systemic infection [69].

We recognise the sampling unevenness amongst this patient cohort. However, detection of multiple sequence types of *C. albicans* and *C. dubliniensis* in patients who were sampled on a single occasion, and at the same time detection of a single sequence type in patients who were sampled at different time points, suggests that detection of multiple sequence types is not dependent on the number of serial sampling events in the same patient.

*C. albicans* isolates not in clade 1 were nearly exclusively retrieved from oral candidosis patients. Overall, we detected higher clonal diversity in patients presenting without clinical evidence of oral mucosal candidosis but with risk factors for oral candidosis. On the other hand, patients yielding a single diploid strain type from clade 1 *C. albicans* presented with clinical oral candidosis. This interesting observation is in keeping with the reduced microbial diversity seen in association with oral inflammation [70]. However, associations between infective states and clonal variability levels cannot be derived from this study. Clearly, a much larger cohort would be required to implement statistical assertions as to associations of *Candida* spp. and/or strain types with particular clinical traits.

Computational deconvolution of the haplotypic phase among populations of micro-variants was used here with MLST datasets to implicate founder population genetic types and assist in identifying progenitor strains types. These data highlight the potential for high-resolution sampling strategies combined with novel analytical approaches to make inferences when sampling longitudinally from a clinically diverse cohort.

Improved ability to make more in-depth analyses of *Candida* molecular epidemiology can be attained using empirically-derived phased sequence data from long-read, high-throughput MLST strategies [71,72] and genome-wide yeast and fungal population studies [73–77]. While we did not find evidence of recombination between different clades, as recently disclosed for collections of *C. glabrata* and *C. albicans* derived from a variety of niches and geographical areas [63,78], future genome-wide studies of oral isolates will determine the true extent of genomic rearrangements and aneuploidy.

Future studies using a combination of metagenetic, population genomic and host genotype sequencing, with systematic sampling strategies and large-scale phenotypic analysis will improve our ability to understand the co-evolution of oral pathogens such as *Candida* spp. in the context of poly-species biofilm in health and disease. Longitudinal studies of the population structure of *Candida* spp., such as the current study, are few and continue to contribute meaningfully alongside emerging high-throughput datasets that also include strain type surveillance [79, 80].

## Supplementary Material

Supplemental Material
