# Supplementary material for: Clonal evolution of Candida albicans, Candida glabrata and Candida dubliniensis at oral niche level in health and disease
Source: J Oral Microbiol. 2021 Mar 15;13(1):1894047. doi: 10.1080/20002297.2021.1894047 (PMC7971237; doi:10.1080/20002297.2021.1894047)
Supplement: Supplemental Material [file ZJOM_A_1894047_SM4511.zip › Supplementary files/Figure.docx]

**Supplementary Material**

**Figure S1: Phased haplotypes of microvariants.**


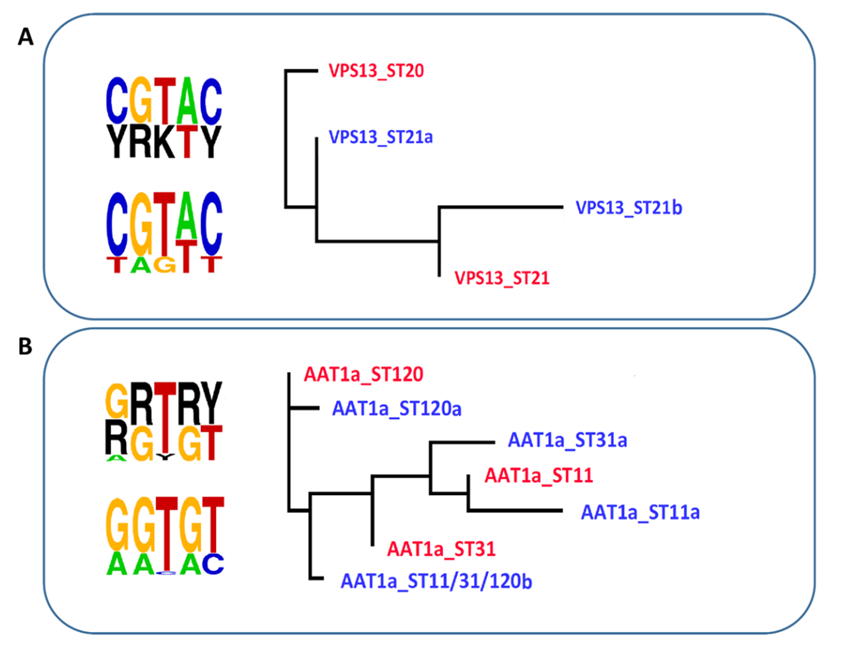
Cladogram (right) of P13 VPS13 and AAT1a microvariant sequences including the unphased ST (red) and both phased deconvolved sequences (blue) and nucleotide motif logos (left) for the unphased ST nucleotide variants (top) and phased haplotypes (bottom)
