# Supplementary material for: Clonal evolution of Candida albicans, Candida glabrata and Candida dubliniensis at oral niche level in health and disease
Source: J Oral Microbiol. 2021 Mar 15;13(1):1894047. doi: 10.1080/20002297.2021.1894047 (PMC7971237; doi:10.1080/20002297.2021.1894047)
Supplement: Supplemental Material [file ZJOM_A_1894047_SM4511.zip › Supplementary files/Table.docx]

**Table S1**. Number of *Candida* spp. strain types identified and clinical features of 20 dental patients

Isolates of *C. albicans*, *C. glabrata* and *C. dubliniensis* were obtained from oral samples from 20 dental patients and sequenced for MLST. The number of strain types identified for each *Candida* spp. are reported. Additional clinical features of patients sampled are included.

| Patient | P1 | P2 | P3 | P4 | P5 | P6 | P7 | P8 | P9 | P10 | P11 | P12 | P13 | P14 | P15 | P16 | P17 | P18 | P19 | P20 |
| --- | --- | --- | --- | --- | --- | --- | --- | --- | --- | --- | --- | --- | --- | --- | --- | --- | --- | --- | --- | --- |
| *C. albicans* |  | 1 | 1 | 1 | 1 |  |  |  | 1 | 2 | 1 | 1 | 3 |  |  | 1 |  | 4 |  | 1 |
| *C. glabrata* | 1 |  |  |  | 1 | 1 |  |  |  |  |  |  |  |  |  |  |  |  |  |  |
| *C. dubliniensis* |  |  |  |  |  |  |  | 2 |  | 2 |  |  |  |  | 1 |  |  |  |  |  |
| Candidosis (erythematous) | Yes | Yes | yes | no | yes | no | no | no | yes | yes | yes | yes | no | no | no | yes | yes | no | yes | yes |
| Dentures | full upper | full upper and lower | full upper | partial lower | partial upper | partial lower | partial lower | partial lower | partial upper | partial upper | partial upper | full upper | none | none | none | full upper, lower partial | partial upper | partial upper and lower | full upper | full upper |
| Other known predisposing factors to candidosis | smoking | smoking | smoking, steroid inhaler | smoking | smoking, steroid inhaler | none | none | none |  | smoking, HIV | drug-induced dry mouth | diabetes, smoking, drug-induced dry mouth | diabetes, smoking, drug-induced dry mouth | none | none | none | smoking, anaemia | none | none | long-term broad-spectrum antibiotics |
| Periodontitis | mild | none (edentulous) | mild | severe | mild | mild | mild | mild | mild | severe | moderate | moderate | mild | none | severe | severe | mild | severe | mild | mild |
